# Exchangeable APOA1 on HDL inhibits LDL binding to proteoglycans

**DOI:** 10.1016/j.jlr.2025.100885

**Published:** 2025-08-21

**Authors:** Esmond N. Geh, Debi K. Swertfeger, Isabella Roscoe, Scott E. Street, Alexiana Bursey, Hannah Sexmith, Laura A. Woollett, W. Sean Davidson, Amy Sanghavi Shah

**Affiliations:** 1 Division of Endocrinology, Department of Pediatrics, Cincinnati Children's Hospital Medical Center & The University of Cincinnati College of Medicine, Cincinnati, OH, USA; 2 Department of Pathology and Laboratory Medicine, University of Cincinnati, Cincinnati, OH, USA

**Keywords:** HDLs, LDLs, proteoglycans, APOA1

## Abstract

The entrapment of LDLs by proteoglycans (PGs) in the extracellular matrix of the arterial intima is a key initial step in the development of atherosclerosis. HDLs can interfere with this process, but the underlying mechanism is not fully understood. The aim of this study was to investigate the mechanisms by which HDL inhibits LDL binding to PG. An In-Cell ELISA was used to measure the binding of LDL to PGs in the extracellular matrix synthesized by mouse vascular smooth muscle cells. Fast-protein liquid chromatography, immunoprecipitation, SDS-PAGE, and immunoblotting analysis were performed to characterize how HDL and its apolipoproteins inhibit LDL binding to PGs. HDL and APOA1 inhibited LDL binding to PGs in a dose-dependent manner. Competition experiments showed that HDL did not compete directly with LDL for PG binding. Instead, APOA1 dissociated from HDL and associated with LDL, reducing the ability of LDL to bind PGs. This was demonstrated by separating HDL and LDL using porous filters of different sizes and tracking the movement of either HDL or APOA1. When APOA1 was solidly anchored to HDL particles, HDL lost the ability to affect LDL-PG binding. HDL inhibits LDL binding to PGs through an interaction with its main apolipoprotein, APOA1, specifically, a pool of loosely attached, exchangeable, lipid-free APOA1 on the HDL surface. These findings identify lipid-free APOA1 as a critical mediator of the ability of HDL to reduce LDL retention in the arterial wall and provide new insights into the antiatherogenic properties of HDL.

Entrapment of apolipoprotein (APO) B-100 containing LDLs by proteoglycans (PGs) in the extracellular matrix of the arterial intima is a key initial step in the development of atherosclerotic vascular lesions (1). This retention triggers a cascade of events leading to oxidative modification of LDL (2), uptake by macrophages, and subsequent foam cell formation (3) as well as accumulation of extracellular lipids, particularly cholesterol (4, 5). Thus, the interaction between LDL and PGs is considered a crucial step in the initiation and progression of atherosclerosis.

HDLs have long been recognized to exhibit a range of atheroprotective effects, primarily through the process of reverse cholesterol transport, where HDL particles remove excess cholesterol from peripheral tissues and deliver it to the liver for excretion as biliary cholesterol or for conversion into bile acids (6). In addition, HDL has also been shown to possess anti-inflammatory, antioxidant, and antithrombotic effects (7, 8, 9). A more recently highlighted atheroprotective role of HDL is its capacity to interfere with the LDL transcytosis and binding to PGs (10).

HDL particles may reduce LDL retention by directly or indirectly disrupting LDL-PG interactions. Camejo *et al.* (11) used an insoluble complex formation assay to show a reduction of LDL-PG insoluble complex formation as the levels of HDL in serum increased. This inhibitory effect of HDL was attributed to its protein component, since exogenous addition of APOA1 showed a similar decrease of LDL-PG complex formation. HDL not only had the ability to disrupt LDL-PG complex formation but also solubilize preformed complexes, suggesting a potential antiatherosclerotic role in vivo [5, 8]. These observations were later followed up by Umaerus *et al.* (12) who postulated that the ability of HDL particles to prevent LDL-PG binding was mediated by APOE molecules on HDL particles. APOE is well known to interact with PGs through a specific binding site and was therefore proposed to compete for LDL binding to PG, thus reducing the accumulation of LDL (13).

Despite these initial observations, the precise molecular and biochemical mechanisms involved in the ability of HDL to inhibit LDL-PG interactions remain poorly defined. The aim of this study is to investigate the molecular basis of HDL-mediated inhibition of LDL binding to PGs and thus provide a new insight into the interplay between lipoproteins in the vessel wall and atherogenesis.

## Materials and methods

### Reagents and antibodies

Biotinylated Goat Anti-Human APOB-100 and HRP-conjugated goat anti-human APO B antibodies were purchased from Academy Bio-Medical, Houston, TX. Chondroitin sulfate (CS) monoclonal antibody (CS-56) and HRP-conjugated goat anti-rabbit IgG were obtained from Life Technologies, Carlsbad, CA. ELISA-Bright HRP substrate and 96-well plates were obtained from VWR International, Batavia, IL. Casein was from EMD Millipore, Temecula, CA, and G418 (Geneticin) was from InvivoGen, San Diego, CA. Carbohydrate Coupling Resin (Hydrazine) and HOOK-NHS Biotin were from G-Biosciences, St Louis, MO and UltraLink Biosupport was from Thermo Scientific. Goat Anti-Human APOA1, Goat Anti-Human APOA2, Goat Anti-Human APOE, and Anti-Human APOB-100 Sepharose 4B™ Gel were purchased from Fortis Life Sciences, Boston, MA. The Phospholipids C kit was purchased from Wako Diagnostics, Osaka, Japan, and the Fast Micro Equilibrium Dialyzer was purchased from Harvard Apparatus, Holliston, MA.

### Cell culture and maintenance

Mouse vascular (aortic) smooth muscle (MOVAS) cell line was purchased from ATCC (Manassas, VA) and cultured in HyClone DMEM/high glucose supplemented with 10% FBS, penicillin (100 units/ml), streptomycin (100 μg/ml), and 200 μg/ml of G418. The media were changed twice a week, and the cells were split at a 1:6 ratio when they reached 90% confluence. For In-Cell ELISA (ICE) experiments, the cells were seeded at 25,000 cells/well in 96-well polystyrene plates. Cells were harvested for experiments when they were 3–5 days post confluent.

### Lipoprotein isolation

LDL and HDL were isolated by sequential ultracentrifugation from plasma obtained from Hoxworth Blood Bank, as previously described (14). Protein concentrations were determined using the BCA method with the Pierce BCA protein determination kit.

### Size-exclusion chromatography

In some experiments, LDL isolated by size-exclusion chromatography (SEC) was used (15). Fractions corresponding to APOB-containing LDL were collected, pooled, and concentrated using Amicon Ultra-4 10K centrifugal columns (MilliporeSigma, Burlington, MA).

### ICE inhibition assay (LDL-PG binding inhibition)

An ICE to quantify APOB bound to PGs was performed as described (16).

All inhibition experiments were conducted using equal amounts (by protein mass) of LDL and HDL. To assess LDL-PG binding inhibition, LDL (1 mg/ml) and HDL (or APOA1, APOA2, and APOE) were pipetted into tubes and incubated at room temperature for 15 min. Following incubation, ICE was performed.

### Gel electrophoreses and Western blot analyses

SDS-PAGE analyses were performed as described (17). For Blue Native (BN) PAGE analysis, NativePAGE Bis-Tris gels from Invitrogen (Carlsbad, CA) were used according to the manufacturer's protocol. For Western blot (immunoblot) analysis, proteins were transferred onto nitrocellulose membranes overnight using a wet-transfer protocol.

### Preparation of reconstituted HDL particles and POPC vesicles

POPC (Avanti Polar Lipids, Alabaster, AL) reconstituted HDL (rHDL) particles were prepared at a lipid-to-recombinant APOA1 molar ratio of 85:1, following the method of Jonas (18). The lipids were dried under nitrogen and resuspended in Tris buffer. Deoxycholate was then added, and the solution was incubated in a 39 °C water bath for 1 h, with gentle vortexing every 15 min. Recombinant APOA1 was subsequently added, and the mixture was incubated at 37 °C for 1 h. The cholate was removed by dialysis against standard PBS, with four buffer changes of 4 l each. Particle analysis was performed using BN-PAGE.

To prepare small unilamellar vesicles (SUVs), 1 ml of POPC lipids (60 mg/ml) was dispensed into a glass borosilicate tube. The chloroform was evaporated under a gentle stream of nitrogen in a fume hood until completely dry. The tube was placed in a SpeedVac for an additional 30 min to ensure complete removal of solvent. Next, 1 ml of PBS was added to the dried lipid film, and the mixture was vortexed and sonicated for 1 h. The resulting solution was applied to a Superose 6 increase gel filtration column pre-equilibrated with PBS and run at a flow rate of 0.5 ml/min. The major peak appeared after the void volume was collected, and the phospholipid concentration was determined using a Phospholipids C kit, according to the manufacturer's protocol.

### Immunoprecipitation and pull-down

Immunoprecipitation was performed using antibody-crosslinked beads following standardized methods. For pull-down assays, CS and LDL were coated onto hydrazine and UltraLink beads, respectively, using the manufacturer's protocol (see *Reagents and antibodies* subsection).

### Immunoaffinity chromatography

For immunoaffinity chromatography, fast-protein liquid chromatography columns were packed with approximately 5 ml of anti-APOA1, anti-APOA2, anti-APOE, or anti-APOB immunoaffinity resins obtained from Genprice, Inc, San Jose, CA. One milliliter of 2 mg/ml LDL (or HDL) was injected into the column, and the column was equilibrated with PBS (buffer A) at 0.5 ml/min until a total volume of 0.5 CV was reached. After equilibration, the flow rate was reduced to 0.3 ml/min to facilitate binding. This was followed by a PBS wash and elution with 3 M sodium thiocyanate (buffer B). Peak fractions were collected, desalted using 10 ml desalting columns, and concentrated using a 10 kDa molecular weight cutoff Amicon centrifugal filter.

### Biotin labeling and paraformaldehyde crosslinking

Biotin labeling was performed using the HOOK-NHS Biotin Labeling Kit (G-Biosciences), following the manufacturer's protocol. HDL crosslinking was carried out by incubating 1 ml of 4 mg/ml HDL with 4% paraformaldehyde (PFA) for 20 min. Following incubation, the mixture was desalted using 5 ml desalting columns (SpinOUT™ GT-100; G-Biosciences) according to the manufacturer's instructions. The desalted solution was then further purified using a Superdex Increase SEC column.

### Equilibrium dialysis

Equilibrium dialysis was performed using a Fast Micro Equilibrium Dialyzer device with prewetted membranes (300, 100, and 10 kDa cutoffs) from Harvard Apparatus. The membranes were briefly rinsed with double-distilled water and then equilibrated in a 6-well plate containing PBS for 20 min. After equilibration, the membrane was sealed between the two chambers by tightly screwing them together. Next, 100 μl of PBS or 1 mg/ml of LDL was pipetted into one chamber and sealed with its cap. The microdialyzer was then inverted, and 100 μl of 1 mg/ml HDL was added to the second chamber. The device was placed in a horizontal position such that liquid in each chamber was in contact with the membrane and incubated for 16 h. After incubation, the caps were removed, and the samples were retrieved, and stored at 4 °C until use.

### Statistical analysis

All data are reported as the sample mean ± standard deviation. An unpaired Student's *t*-test was used in all our statistical analyses, and a *P* value of <0.01 was considered statistically significant.

## Results

### HDL decreases LDL binding to PGs in MOVAS cells

**Fig. 1:**
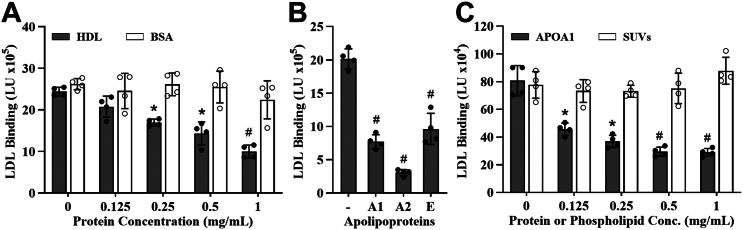
HDL inhibits LDL binding to PGs. ICE inhibition assay showing the effect of preincubating LDL with (A) increasing concentrations of HDL (*gray bars*) or BSA (*white bars*), (B) HDL apolipoproteins (APOA1, APOA2, and APOE), and (C) increasing concentrations of APOA1 (*gray bars*) or SUVs (*white bars*). Results are expressed as LDL binding in light units (LU). Data represent the mean ± standard deviation (n = 4). An unpaired *t*-test showed a statistically significant difference of ∗*P* < 0.001 and #*P* < 0.0001. A1, APOA1; A2, APOA2; E, APOE; conc., concentration.

Previous studies have shown that HDL inhibits LDL binding to PGs in various in vitro systems (11, 12, 19). To assess this effect in our cell culture model, we incubated MOVAS cells with 1 mg/ml of LDL protein preincubated as previously described (16) with varying concentrations of human HDL isolated by ultracentrifugation. As shown in Fig. 1A (*black bars*), LDL-PG binding decreased with increasing HDL concentrations, suggesting that HDL inhibits LDL binding to PGs in MOVAS cells. In contrast, treatment with purified BSA failed to inhibit LDL-PG binding (Fig. 1A, *white bars*).

Given prior work by Camejo *et al.* (11) and others (12), we evaluated whether all HDLs' most abundant APOs (APOA1, APOA2, and APOE) also inhibited LDL binding to PGs. Figure 1B shows that APOA1, APOA2, and APOE all inhibited LDL binding to PGs, with APOA2 exhibiting the greatest potency when assessed at equal protein concentrations, 1 mg/ml.

Next, we asked whether phospholipids that mimic those present in HDL play a role in its inhibitory effect on LDL-PG binding. To test this, we generated SUVs using POPC lipids and examined their effect on LDL-PG binding at various concentrations in MOVAS cells. As shown in Fig. 1C, while APOA1 inhibited LDL-PG binding in a dose-dependent manner, POPC SUVs had no effect at any concentration, suggesting that the inhibitory effect of HDL is mediated by its protein component, specifically apolipoproteins.

### HDL inhibition of LDL-PG binding is not mediated by APOA2 or APOE

**Fig. 2:**
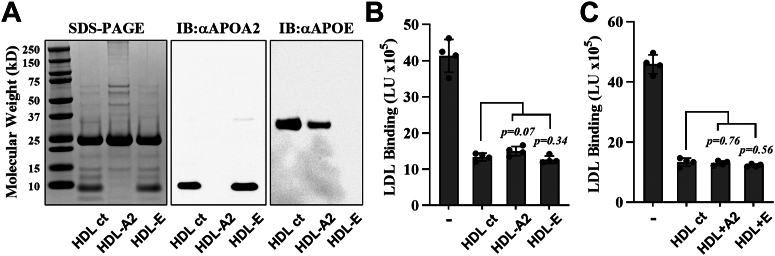
HDL inhibition of LDL-PG binding is not mediated by APOA2 or APOE. (A) SDS-PAGE analysis and corresponding immunoblots (IBs) using antibodies against APOA2 (αAPOA2) and APOE (αAPOE) confirm the immunodepletion of APOA2 and APOE from HDL. B: ICE inhibition assay showing the effect of LDL incubation with HDL immunodepleted of APOA2 and APOE. C: ICE inhibition assay assessing LDL binding after the reintroduction of APOA2 and APOE into HDL. Results are expressed as LDL binding in light units (LU). Data represent the mean ± standard deviation (n = 4). An unpaired *t*-test showed no significant difference between intact and immunodepleted HDL. A2, APOA2; ct, control; E, APOE; IB, immunoblot.

To assess the relative contributions of HDL's major apolipoproteins to inhibit LDL-PG binding, we immunodepleted APOA2 and APOE from ultracentrifugally isolated HDL. Western blot analysis confirmed successful immunodepletion of APOA2 and APOE (Fig. 2A). As shown in Fig. 2B, immunodepletion of either APOA2 or APOE did not affect the ability of HDL to inhibit LDL-PG binding. ELISA measurements indicated that the initial HDL samples contained APOA2 and APOE at concentrations of 0.1 mg/ml and 0.001 mg/ml, respectively (data not shown). To further investigate the contributions of these apolipoproteins, we added exogenous APOA2 or APOE to HDL, resulting in an approximate doubling of each apolipoprotein to HDL. The results (Fig. 2C) show that this supplemental addition of APOA2 or APOE had no effect on the ability of HDL to inhibit LDL-PG binding.

### APOA1 binds LDL and interferes with LDL-PG binding

**Fig. 3:**
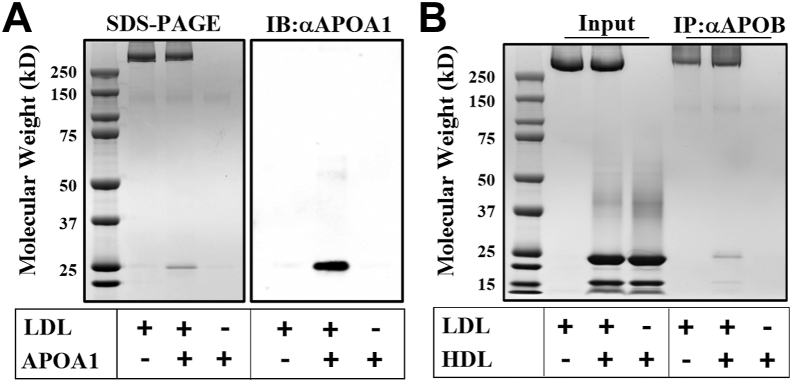
APOA1 associates with LDL in vitro. Beads coated with an APOB antibody were used to pull down LDL, LDL preincubated with APOA1 (A), or HDL (B). SDS-PAGE and immunoblot with an APOA1 antibody show LDL and APOA1 complexes coimmunoprecipitated using the APOB antibody.

Competition experiments indicated that HDL does not directly compete with LDL for binding to cell PGs (not shown), leading us to hypothesize that HDL modifies LDL's PG affinity through an interaction mediated by its major apolipoprotein, APOA1. To test this hypothesis, we performed a coimmunoprecipitation experiment in which lipid-free APOA1 (1 mg/ml) was incubated with LDL (1 mg/ml) for 15 min. The resulting solution was immunoprecipitated using an anti-APOB antibody, and a Western blot was performed using an anti-APOA1 antibody. As shown in the Coomassie-stained SDS-PAGE gel in Fig. 3A, immunoprecipitation of LDL alone with the anti-APOB antibody recovered APOB (band at the top of the gel) but did not pull-down APOA1. When APOA1 and LDL were incubated together, the anti-APOB pull-down also included a band consistent with APOA1, which was confirmed by Western blot against APOA1. This indicates that a portion of lipid-free APOA1 is capable of binding LDL. Similar results were obtained when HDL isolated by ultracentrifugation was used instead of APOA1 (Fig. 3B).

Next, we assessed the functional significance of the APOA1-LDL interaction using a pull-down assay with CS-coated beads. We hypothesized that if APOA1 reduces LDL-PG binding, preincubating LDL with APOA1 before adding it to the CS beads should result in a decrease of the amount of bound LDL and increase the unbound fraction. Consistent with this hypothesis, the results showed a higher amount of APOB in the unbound fraction (Supplemental Fig. S1, *red arrow*) when APOA1 was present compared with LDL alone, indicating that APOA1 reduces LDL binding to CS.

**Fig. 4:**
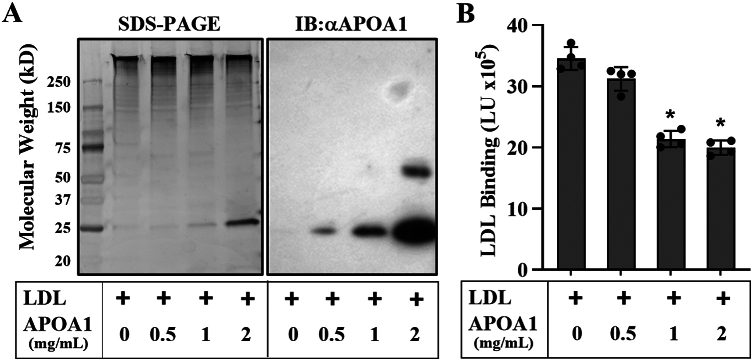
APOA1 interferes with LDL binding to PGs. A: Silver-stained gel and Western blot images of LDL fractions repurified after preincubation with increasing concentrations of APOA1. B: ICE assay performed using the repurified fractions from A. Results are expressed as LDL binding in light units (LU). Data represent the mean ± standard deviation (n = 4). An unpaired Student's *t*-test indicated statistical significance (∗*P* < 0.001). αAPOA1, APOA1 antibody; IB, immunoblot.

To validate these findings in our cell culture system, we incubated lipid-free APOA1 with LDL for 15 min in increasing concentrations, then separated the mixture using SEC. The LDL-containing fractions were collected, pooled, analyzed by SDS-PAGE, and used in a PG binding assay in MOVAS cells. As shown in Fig. 4A, increasing APOA1 levels resulted in a proportional increase in APOA1 association with LDL. This increase was accompanied by a corresponding decrease in LDL binding to PGs in MOVAS cells (Fig. 4B). These results suggest that when APOA1 is associated with LDL, it reduces the ability of LDL to bind PG.

### “Resident” APOA1 on LDL does not affect LDL-PG binding

**Fig. 5:**
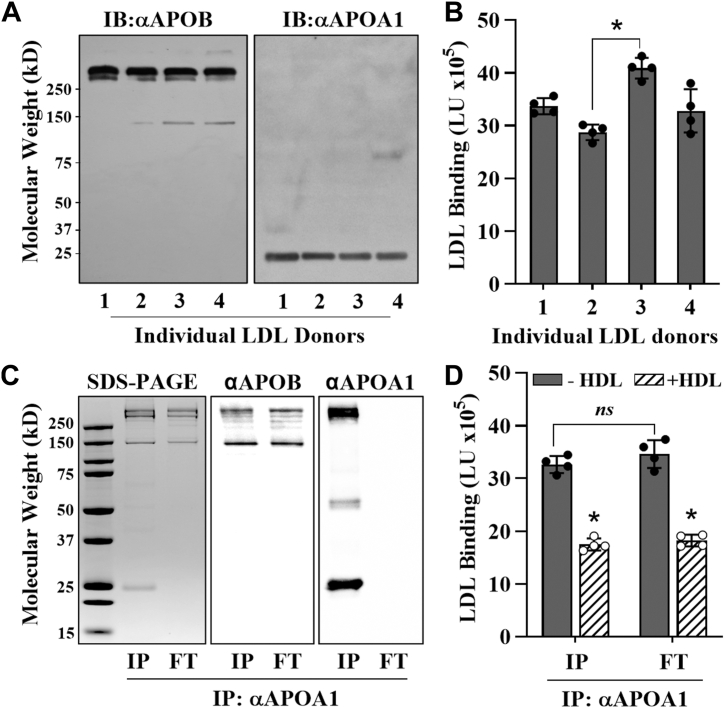
Resident APOA1 on LDL does not affect LDL binding to PGs. A: Immunoblots (IBs) of fast-protein liquid chromatography—purified LDL from plasma of various donors using antibodies against APOB (αAPOB) and APOA1 (αAPOA1). B: LDL-PG binding assays performed using the LDL samples from A. C: SDS-PAGE and Western blot images of ultracentrifugally isolated LDL, separated into APOA1-containing (IP) and APOA1-depleted (FT) fractions using anti-APOA1 antibody. D: ICE inhibition assays performed using the LDL samples from C. Results are expressed as LDL binding in light units (LU). Data represent the mean ± standard deviation (n = 4). An unpaired Student's *t*-test indicated statistical significance (∗*P* < 0.001).

APOA1 is known to be present on LDL in vivo, and the amount of APOA1 associated with LDL is elevated in patients with coronary artery disease (20). One interpretation of these findings is that APOA1-associated LDL may represent an adaptive response to atherosclerosis, which could explain its elevated levels in these patients. Based on this report and our findings above, we hypothesized that the binding of LDL to PG will be inversely proportional to the amount of APOA1 that is associated with LDL in human plasma samples. To test this, we collected plasma from four human donors and fractionated their LDL by SEC. The anti-APOB immunoblot shown in Fig. 5A confirms equivalent LDL loading (2 μg). Densitometry analysis of the Western blot image using an APOA1 antibody revealed variability in APOA1 levels associated with LDL among individuals. Donor 1 exhibited approximately one-third more APOA1 than donor 4 as determined by densitometry, respectively. Surprisingly, the LDL-PG binding assay results showed no significant correlation between APOA1 content that is associated with LDL and the PG binding of the particles (Fig. 5B). Analysis of LDL from 9 other donors yielded similar results (not shown). These findings suggest that “resident” APOA1, that is, the amount of APOA1 that has been associated with LDL for a significant amount of time, does not play a role in inhibiting LDL-PG binding. However, APOA1 introduced in a brief time frame can affect LDL-PG binding.

Next, we investigated how the presence of resident APOA1 in LDL affects the ability of HDL to inhibit LDL-PG binding. To this end, we performed APOA1 immunoaffinity chromatography on isolated LDL to separate it into APOA1-containing and APOA1-depleted fractions. SDS-PAGE analysis, along with Western blotting using anti-APOB and anti-APOA1 antibodies, confirmed the successful immunodepletion of APOA1 in the depleted fraction (Fig. 5C). As shown in Fig. 5D, PG binding did not differ significantly between the two fractions, supporting the idea that resident APOA1 does not affect LDL-PG interactions. However, HDL was still able to reduce LDL-PG binding in both cases. These results suggest that while resident APOA1 has no effect on PG binding, APOA1 transferred from HDL reduces LDL-PG binding regardless of the presence of resident APOA1.

### Lipid-bound APOA1 does not inhibit LDL-PG binding

**Fig. 6:**
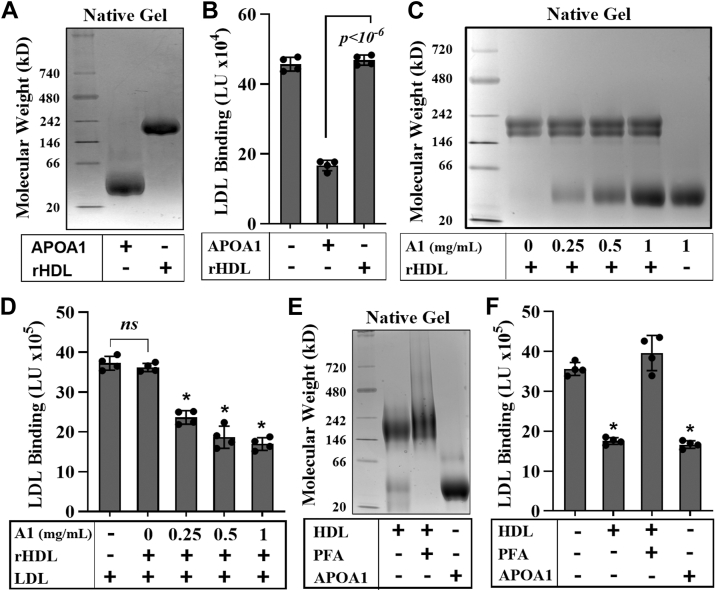
Lipid-associated APOA1 does not inhibit LDL-PG binding. BN gel analysis and ICE inhibition assays evaluating the effect of POPC-rHDL particles (A and B), increasing concentrations of APOA1 titrated into rHDL (C and D), and native HDL crosslinked with PFA (E and F), on LDL-PG binding inhibition. Results are expressed as LDL binding in light units (LU). Data represent the mean ± standard deviation (n = 4). An unpaired Student's *t*-test indicated statistical significance (∗*P* < 0.001). A1, APOA1.

The results above suggest that HDL must be present to provide a source of APOA1 that interacts with LDL, thereby limiting its ability to bind PGs. Ultracentrifugally isolated HDL, when electrophoresed on a BN gel, separates into two distinct APOA1 populations: lipid-bound and lipid-free APOA1 (Supplemental Fig. S2). In contrast, purified rHDL as well as HDL crosslinked with 4% PFA lacks a lipid-free pool of APOA1. To further explore whether it is the lipid-bound or lipid-free APOA1 associated with HDL that inhibits LDL-PG binding, we generated rHDL particles using POPC lipid and APOA1, purified the rHDL away from free APOA1 using SEC, and tested its ability to inhibit LDL-PG binding. As shown in Fig. 6A, rHDL exhibited a tight band with no significant lipid-free population. ICE inhibition experiments performed with rHDL particles showed that while free APOA1 inhibited LDL-PG binding, rHDL without lipid-free APOA1 did not (Fig. 6B). We then investigated whether adding increasing amounts of lipid-free APOA1 to rHDL particles could restore their ability to inhibit LDL-PG binding. Indeed, a dose-dependent addition of APOA1 to rHDL particles (Fig. 6C) resulted in a corresponding decrease in LDL-PG binding (Fig. 6D).

Next, we asked whether crosslinking ultracentrifugally isolated HDL and essentially eliminating the lipid-free APOA1 would reduce its ability to inhibit LDL binding to PGs as seen with rHDL. We crosslinked ultracentrifugally isolated HDL using 4% PFA for 20 min, followed by desalting and purifying over a single Superdex fast-protein liquid chromatography column to remove any residual-free APOA1. The BN gel results (Fig. 6E) showed that while control HDL displayed two prominent bands, PFA-treated HDL displayed only the lipid-associated APOA1 population and lacked the lipid-free form. Functional characterization by ICE inhibition revealed that while control HDL inhibited LDL-PG binding, the PFA-treated crosslinked HDL did not (Fig. 6F). Taken together, these results point to lipid-free APOA1 and not the lipid-bound APOA1 on HDL as a necessary component for native HDL to inhibit LDL-PG binding.

### Lipid-free APOA1 is required for HDL to inhibit LDL-PG binding

**Fig. 7:**
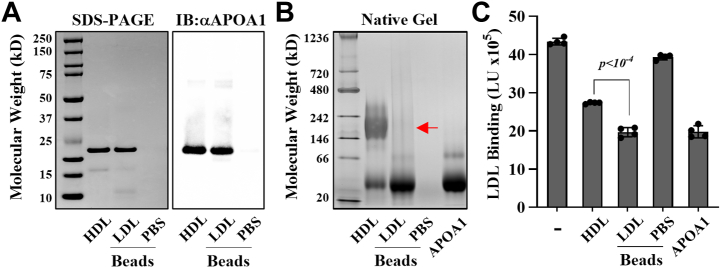
HDL-derived lipid-free APOA1 inhibits LDL-PG binding. (A) SDS-PAGE and immunoblot analysis, (B) BN-PAGE analysis, and (C) ICE inhibition assay of eluates obtained from LDL-coated or PBS-coated control (Ctrl) beads following incubation with HDL. LDL binding is presented in light units (LU). Data are shown as mean ± standard deviation (n = 4). Statistical analysis using an unpaired Student's *t*-test revealed a significant difference (∗*P* < 0.0001) between lipid-free APOA1 and HDL. Ctrt, control.

To determine whether the transfer of APOA1 from HDL to LDL is required for HDL-mediated inhibition of LDL-PG binding, HDL was incubated with either LDL-coated beads or control beads lacking LDL. After incubation, bound proteins were eluted and analyzed by SDS-PAGE and Western blotting. As shown in Fig. 7A, APOA1 was detected in the eluate from LDL-coated beads, but not from control beads, indicating that APOA1 had transferred from HDL to LDL. Native PAGE analysis of the eluates confirmed that the transferred APOA1 migrated as a single band corresponding to lipid-free APOA1, with no detectable lipid-associated forms (Fig. 7B, *red arrow*). ICE inhibition assays showed that this APOAI eluted off the LDL-coated beads was as effective as recombinant APOA1 in inhibiting LDL-PG binding and was more effective than HDL at equal concentrations of APOA1 (Fig. 7C). These results indicate that lipid-free APOA1, derived from HDL, can transfer to LDL and inhibit its binding to PGs, suggesting a dynamic exchange of a functional, lipid-free pool of APOA1 between HDL and LDL.

To determine whether the inhibition of LDL-PG binding is mediated by HDL-associated (lipid-bound) APOA1 or by lipid-free APOA1, we performed equilibrium dialysis using membranes with molecular weight cutoffs of 300, 100, and 10 kD. We hypothesized that lipid-bound APOA1, because of its larger size, would cross the 300 kD membrane but not the 100 or 10 kD membranes, whereas lipid-free APOA1 would traverse both the 300 and 100 kD membranes but not the 10 kD membrane. The dialysis was performed using 100 μl of 1 mg/ml LDL on one side of the membrane and 100 μl of 1 mg/ml HDL labeled with a fluorescent phospholipid (phosphatidylethanolamine-phosphatidylcholine; 1,2-dioleoyl-*sn*-glycero-3-phosphoethanolamine-*N*-carboxyfluorescein) on the opposite side (see *Materials and Methods* section).

**Fig. 8:**
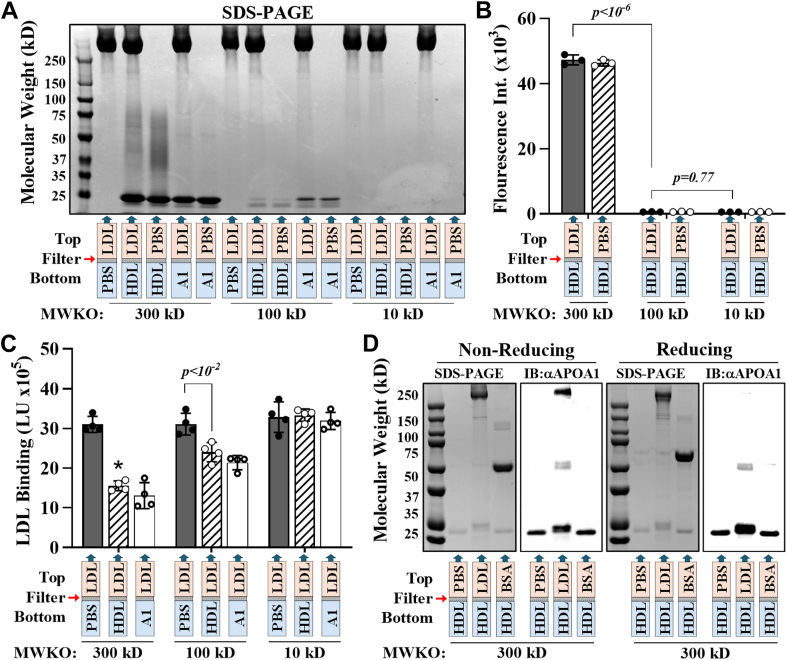
Lipid-free APOA1 is transferred from HDL to LDL during equilibrium dialysis. SDS-PAGE analysis (A) and fluorescence intensity measurements (B) were performed on LDL and PBS dialysates (chamber 1, *top*) obtained following equilibrium dialysis against HDL labeled with a fluorescent phospholipid or recombinant lipid-free APOA1 (chamber 2, *bottom*). An unpaired Student's *t*-test indicated a statistical significance difference (*P* < 10^4^) between the 300 and 100 kD membranes in fluorescent intensities. ICE inhibition assay results using LDL from the top chamber (C) are also shown expressed as LDL binding in light units (LU). Data represent the mean ± standard deviation (n = 4). An unpaired Student's *t*-test indicated a statistical significance difference (*P* < 10^2^). D: SDS-PAGE and Western blot analysis of PBS, LDL, and BSA dialysates using antibody against APOA1 (αAPOA1).

**Fig. 9:**
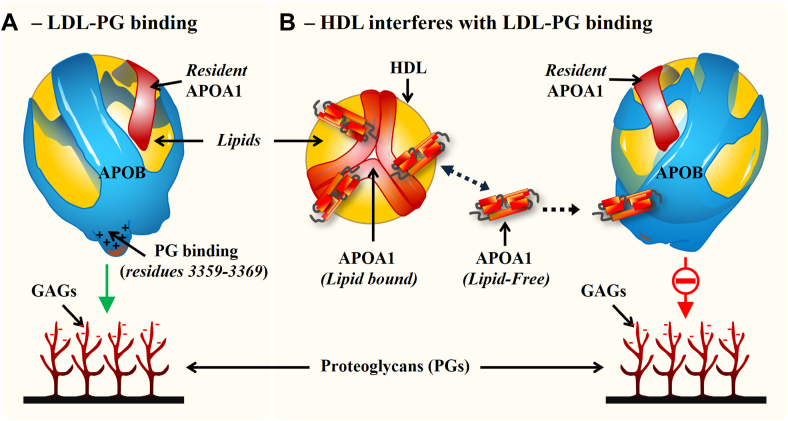
Schematic depicting the proposed mechanism of HDL inhibition of LDL-PG binding. A: LDL binds freely to PGs through an interaction of positively charged lysine residues on APOB with the negatively charged glycoaminoglycan (GAG) chains on proteoglycans. B: Loosely associated, lipid-free APOA1 dissociates from HDL particles, binds to LDL, altering the conformation of APOB, thereby preventing LDL from binding to proteoglycans. The “resident APOA1” on LDL and lipid-bound APOA1 on HDL particles are not involved in this interaction.

As shown in Fig. 8A (protein) and 8B (phospholipid), both APOA1 and phospholipids moved through the 300 kD membrane, but only lipid-free APOA1 moved through the 100 kD membrane, and neither phospholipid or lipid-free APOA1 moved through the10 kD membrane, indicating that lipidated HDL can only move through the 300 kD membrane, whereas lipid-free APOA1 can pass through the 100 kD membrane, and neither can pass through the 10 kD membrane. Furthermore, ICE inhibition analysis demonstrated that this lipid-free APOA1 that passed through the 100 kD membrane, derived from native HDL, retained the ability to inhibit LDL binding to PG *(*Fig. 8C). Similar results were obtained when 1 mg/ml of recombinant APOA1 was dialyzed against 1 mg/ml of LDL using 300 and 100 but not 10 kD membranes (Fig. 8A–C). Interestingly, SDS-PAGE and Western blot analyses of the dialysates, performed under nonreducing and reducing conditions, revealed significantly greater APOA1 transfer across the membranes when LDL was present compared with PBS or BSA (Fig. 8D). Moreover, most APOA1 that crosses the membrane appears to be associated with LDL, as this association is lost under reducing conditions. Native PAGE further revealed a higher concentration of lipid-free APOA1 in dialysates against LDL than against PBS (Supplemental Fig. S3, *red rectangles*), indicating that this lipid-free form has a stronger affinity for LDL than for PBS buffer. Collectively, these findings support a model in which HDL inhibits LDL-PG binding by transferring a pool of loosely associated, lipid-free APOA1 onto LDL, which in turn prevents LDL from interacting with PGs (Fig. 9).

## Discussion

Our current study provides new insights into the molecular mechanisms by which HDL inhibits LDL binding to PGs, a key step in the development of atherosclerosis. HDL inhibition is primarily mediated through APOA1, which directly interacts with LDL to reduce its PG binding. However, not all forms of APOA1 contribute equally to this inhibition. In fact, it is only APOA1 loosely associated with HDL that can transfer to LDL and reduce its PG binding. Resident APOA1 already present on LDL, as well as APOA1 incorporated into rHDL or crosslinked to prevent transfer, does not affect this interaction and is unable to inhibit LDL-PG binding. These results highlight a critical role of lipid-free, exchangeable APOA1 in mediating the atheroprotective function of HDL by reducing LDL retention in the arterial wall.

The inhibition of LDL-PG binding by HDL is not a new concept. Previous studies have shown that HDL and APOA1 interfere with LDL binding to PGs in various in vitro (11, 12, 19) and in vivo systems (21, 22). APOA1 exchangeability is an indicator of atherosclerosis and, when diminished, is associated with greater cardiovascular risk (23). In general, this relationship has been most frequently tied to cholesterol efflux because lipid-free (or poor) forms of APOA1 interact most efficiently with cell surface ABCA1 to form nascent HDL particles and initiate reverse cholesterol transport (24). Additionally, the binding of lipid-free APOA1 to ABCA1 has been shown to reduce the secretion of proinflammatory cytokines, highlighting a potential anti-inflammatory role for lipid-free APOA1 (25). This selective interaction between lipid-free APOA1 and ABCA1 parallels our findings that LDL also preferentially interacts with lipid-free rather than lipid-bound APOA1. It is plausible that these three atheroprotective functions of lipid-free APOA1—cholesterol efflux, anti-inflammatory signaling, and inhibition of LDL-PG binding, operate through a shared mechanism by which HDL exerts its beneficial effects. Further work is needed to study the combinations of these HDL effects in vivo to confirm this hypothesis.

In our hands, a pool of exchangeable APOA1 appears to be responsible for the PG binding inhibition of HDL samples. The idea that APOA1 exists in multiple conformations has been around for quite some time, at least on HDL. Lund-Katz *et al.* (26) used NMR to show that APOA1 on human HDL adopts two major forms: *i*) an integral form in an extended and highly lipid-engaged arrangement that provides structural organization to the HDL particle and *ii*) a transient form that likely resembles an unfolded four-helix bundle that is capable of rapid exchange. Our studies show that when APOA1 is tethered to the HDL particles either by chemical crosslinking or careful isolation of stable rHDL particles away from any lipid-free APOA1, the ability to inhibit LDL binding to PGs is lost. Given recent revelations about the antiatherogenicity of such exchangeable APOA1, more study is needed to determine the conditions, which result in increased APOA1 exchange dynamics.

We also show that APOE and, to a greater extent, APOA2 inhibit LDL-PG binding at concentrations similar to APOA1, suggesting that other HDL apolipoproteins may modulate LDL-PG interactions. However, given the lower plasma concentrations of APOE (∼0.03 mg/ml) and APOA2 (∼0.24 mg/ml) compared with APOA1 (∼1.13 mg/ml) (27), their overall impact in vivo is likely much smaller. In addition, APOA2 has limited exchangeability in plasma because of its high affinity for lipids (28). While our immunodepletion experiments indicate that APOA1 is the primary mediator of the inhibitory effect of HDL on LDL-PG binding, they do not entirely rule out contributions from other HDL-associated apolipoproteins. For example, in APOA1 knockout mice, APOA2 and APOE expressions are significantly increased (29), suggesting these apolipoproteins may compensate for APOA1 in preventing LDL-PG binding. Regarding APOE, Umaerus *et al.* (12) proposed that HDL may inhibit LDL-PG binding by competing with LDL for PG-binding sites, based on data showing that enriching HDL particles with APOE reduces LDL-PG complex formation. In contrast, our data indicate that HDL inhibits LDL-PG binding not by interacting with PGs directly but by transferring lipid-free APOA1 to LDL. Further studies are needed to clarify how APOA2, APOE, and other HDL-associated apolipoproteins influence LDL-PG interactions.

LDL containing resident, or stably incorporated, APOA1 bound PG to the same extent as its APOA1-depleted counterpart, indicating that preassociated APOA1 does not influence LDL-PG interactions. One possible explanation is that APOA1 bound to LDL is associated with lipids, similar to lipid-bound APOA1 on HDL, which remains in close contact with the lipid surface and is therefore unavailable for LDL-PG interactions (30). Another possibility is that the resident APOA1 on LDL is bound to APOB at a site distinct from the PG binding site, or it may simply represent a dysfunctional APOA1 variant crosslinked to LDL, similar to the form found in the vessel wall (31). Regardless, HDL significantly inhibited the binding of both APOA1-associated and APOA1-depleted LDL to PGs. The fact that APOA1-containing LDL can still bind PGs may help explain why APOA1, along with other exchangeable apolipoproteins, is frequently found colocalized with APOB in atherosclerotic vascular lesions (20, 32).

One limitation of our study is that we were unable to correlate the amount of free APOA1 observed on native gels with the functional ICE inhibition assay results. This discrepancy may arise because both techniques capture static snapshots, whereas HDL remodeling is a dynamic process (33, 34, 35). Free APOA1 continuously associates with and dissociates from the HDL lipid core, so its level can vary over time, potentially leading to variability in the native gel results. Another limitation is that LDL binding to PGs is primarily an ionic interaction between the positively charged lysine residues of LDL and the negatively charged sulfate groups of PGs. Lipoprotein properties, such as surface charge, sialic acid content, and APOC3 levels, can influence this interaction. For example, sialic acid residues can alter the net charge of LDL, making it more or less prone to PG binding. The potential effects of such modifications on HDL-LDL interactions were not examined in this study.

In conclusion, our findings uncover the mechanism by which HDL protects against atherosclerosis. HDL transfers a pool of lipid-free, exchangeable APOA1 to LDL, thereby reducing the ability of LDL to bind PGs and, as a result, decreasing its retention in the arterial wall. These findings not only enhance our understanding of the protective role of HDL but also suggest that lipid-free APOA1 could be a key target for therapeutic strategies aimed at boosting the antiatherogenic effects of HDL. Future studies are needed to validate these findings in vivo and to further investigate the molecular details of the APOA1-LDL interaction.

## Data availability

All data supporting the findings of this study are included in the article and supplemental data files.

## Supplemental data

This article contains supplemental data.

## Conflict of interest

The authors declare that they have no conflicts of interest with the contents of this article.

## References

[1] Ference B.A., Ginsberg H.N., Graham I., Ray K.K., Packard C.J., Bruckert E. (2017). Low-density lipoproteins cause atherosclerotic cardiovascular disease. 1. Evidence from genetic, epidemiologic, and clinical studies. A consensus statement from the European Atherosclerosis Society consensus panel. Eur. Heart J..

[2] Steinberg D. (2009). The LDL modification hypothesis of atherogenesis: an update. J. Lipid Res..

[3] Kruth H.S., Huang W., Ishii I., Zhang W.Y. (2002). Macrophage foam cell formation with native low density lipoprotein. J. Biol. Chem..

[4] Lehti S., Nguyen S.D., Belevich I., Vihinen H., Heikkila H.M., Soliymani R. (2018). Extracellular lipids accumulate in human carotid arteries as distinct three-dimensional structures and have proinflammatory properties. Am. J. Pathol..

[5] Grosheva I., Haka A.S., Qin C., Pierini L.M., Maxfield F.R. (2009). Aggregated LDL in contact with macrophages induces local increases in free cholesterol levels that regulate local actin polymerization. Arterioscler Thromb. Vasc. Biol..

[6] Adorni M.P., Ronda N., Bernini F., Zimetti F. (2021). High density lipoprotein cholesterol efflux capacity and atherosclerosis in cardiovascular disease: pathophysiological aspects and pharmacological perspectives. Cells.

[7] Barter P.J., Nicholls S., Rye K.A., Anantharamaiah G.M., Navab M., Fogelman A.M. (2004). Antiinflammatory properties of HDL. Circ. Res..

[8] Brites F., Martin M., Guillas I., Kontush A. (2017). Antioxidative activity of high-density lipoprotein (HDL): mechanistic insights into potential clinical benefit. BBA Clin..

[9] van der Stoep M., Korporaal S.J., Van Eck M. (2014). High-density lipoprotein as a modulator of platelet and coagulation responses. Cardiovasc. Res..

[10] Fung K.Y.Y., Ho T.W.W., Xu Z., Neculai D., Beauchemin C.A.A., Lee W.L. (2024). Apolipoprotein A1 and high-density lipoprotein limit low-density lipoprotein transcytosis by binding SR-B1. J. Lipid Res..

[11] Camejo G., Cortez M.M., Lopez F., Starosta R., Mosquera B., Socorro L. (1980). Factors modulating the interaction of LDL with an arterial lipoprotein complexing proteoglycan: the effect of HDL. Acta Med. Scand. Suppl..

[12] Umaerus M., Rosengren B., Fagerberg B., Hurt-Camejo E., Camejo G. (2012). HDL2 interferes with LDL association with arterial proteoglycans: a possible athero-protective effect. Atherosclerosis.

[13] Hurt-Camejo E., Camejo G., Sartipy P. (1998). Measurements of proteoglycan-lipoprotein interaction by gel mobility shift assay. Methods Mol. Biol..

[14] Melchior J.T., Street S.E., Andraski A.B., Furtado J.D., Sacks F.M., Shute R.L. (2017). Apolipoprotein A-II alters the proteome of human lipoproteins and enhances cholesterol efflux from ABCA1. J. Lipid Res..

[15] Gordon S.M., Deng J., Lu L.J., Davidson W.S. (2010). Proteomic characterization of human plasma high density lipoprotein fractionated by gel filtration chromatography. J. Proteome Res..

[16] Geh E.N., Swertfeger D.K., Sexmith H., Heink A., Tarapore P., Melchior J.T. (2024). A novel assay to measure low-density lipoproteins binding to proteoglycans. PLoS One.

[17] Laemmli U.K. (1970). Cleavage of structural proteins during the assembly of the head of bacteriophage T4. Nature.

[18] Jonas A. (1986). Reconstitution of high-density lipoproteins. Methods Enzymol..

[19] Noma A., Takahashi T., Wada T. (1981). Elastin-lipid interaction in the arterial wall. Part 2. In vitro binding of lipoprotein-lipids to arterial elastin and the inhibitory effect of high density lipoproteins on the process. Atherosclerosis.

[20] Ogasawara K., Mashiba S., Hashimoto H., Kojima S., Matsuno S., Takeya M. (2008). Low-density lipoprotein (LDL), which includes apolipoprotein A-I (apoAI-LDL) as a novel marker of coronary artery disease. Clin. Chim. Acta.

[21] She M.P., Liang P., Huang Y.D., Cai C.B., Ran B.F., Wang Z.L. (1992). HDL and apolipoprotein A1 (apo A1). Their effects on retardation of lipid deposition in aortic intima. Chin Med. J. (Engl).

[22] Ran B.F. (1989). [The inhibitory effect of apolipoproteins in HDL on experimental atherosclerosis in rabbits]. Zhonghua Bing Li Xue Za Zhi.

[23] Borja M.S., Zhao L., Hammerson B., Tang C., Yang R., Carson N. (2013). HDL-apoA-I exchange: rapid detection and association with atherosclerosis. PLoS One.

[24] Borja M.S., Hammerson B., Tang C., Savinova O.V., Shearer G.C., Oda M.N. (2017). Apolipoprotein A-I exchange is impaired in metabolic syndrome patients asymptomatic for diabetes and cardiovascular disease. PLoS One.

[25] Yin K., Deng X., Mo Z.C., Zhao G.J., Jiang J., Cui L.B. (2011). Tristetraprolin-dependent post-transcriptional regulation of inflammatory cytokine mRNA expression by apolipoprotein A-I: role of ATP-binding membrane cassette transporter A1 and signal transducer and activator of transcription 3. J. Biol. Chem..

[26] Lund-Katz S., Liu L., Thuahnai S.T., Phillips M.C. (2003). High density lipoprotein structure. Front. Biosci..

[27] Lin R.C., Miller B.A., Kelly T.J. (1995). Concentrations of apolipoprotein AI, AII, and E in plasma and lipoprotein fractions of alcoholic patients: gender differences in the effects of alcohol. Hepatology.

[28] Saito H., Lund-Katz S., Phillips M.C. (2004). Contributions of domain structure and lipid interaction to the functionality of exchangeable human apolipoproteins. Prog. Lipid Res..

[29] Wang Y., Sawashita J., Qian J., Zhang B., Fu X., Tian G. (2011). ApoA-I deficiency in mice is associated with redistribution of apoA-II and aggravated AApoAII amyloidosis. J. Lipid Res..

[30] Pourmousa M., Song H.D., He Y., Heinecke J.W., Segrest J.P., Pastor R.W. (2018). Tertiary structure of apolipoprotein A-I in nascent high-density lipoproteins. Proc. Natl. Acad. Sci. U. S. A..

[31] DiDonato J.A., Huang Y., Aulak K.S., Even-Or O., Gerstenecker G., Gogonea V. (2013). Function and distribution of apolipoprotein A1 in the artery wall are markedly distinct from those in plasma. Circulation.

[32] Kanter J.E., Bornfeldt K.E. (2021). Apolipoprotein C3 and apolipoprotein B colocalize in proximity to macrophages in atherosclerotic lesions in diabetes. J. Lipid Res..

[33] Curtiss L.K., Valenta D.T., Hime N.J., Rye K.A. (2006). What is so special about apolipoprotein AI in reverse cholesterol transport?. Arterioscler Thromb. Vasc. Biol..

[34] Pownall H.J., Ehnholm C. (2006). The unique role of apolipoprotein A-I in HDL remodeling and metabolism. Curr. Opin. Lipidol..

[35] Rye K.A., Clay M.A., Barter P.J. (1999). Remodelling of high density lipoproteins by plasma factors. Atherosclerosis.

