# Supplementary material for: Exchangeable APOA1 on HDL inhibits LDL binding to proteoglycans
Source: J Lipid Res. 2025 Aug 21;66(9):100885. doi: 10.1016/j.jlr.2025.100885 (PMC12512155; doi:10.1016/j.jlr.2025.100885)
Supplement: Supplemental Information [file mmc1.docx]

**SUPPLEMENTAL INFORMATION:**

**Exchangeable APOA1 on High-density Lipoprotein Inhibits Low-Density Lipoprotein Binding to Proteoglycans**

Esmond N. Geh^1^, Debi K. Swertfeger^1^, Isabella Roscoe^1^, Scott Street^2^, Bursey Alexiana^1^, Hannah Sexmith^1^, Laura A. Woollett^2^, W. Sean Davidson^2^ and Amy Sanghavi Shah^1^.

^1^Division of Endocrinology, Cincinnati Children’s Hospital Medical Center & the Department of Pediatrics, University of Cincinnati College of Medicine, Cincinnati, OH

^2^Department of Pathology and Laboratory Medicine, University of Cincinnati, 2120 East Galbraith Road, Cincinnati, OH 45237-0507, USA.


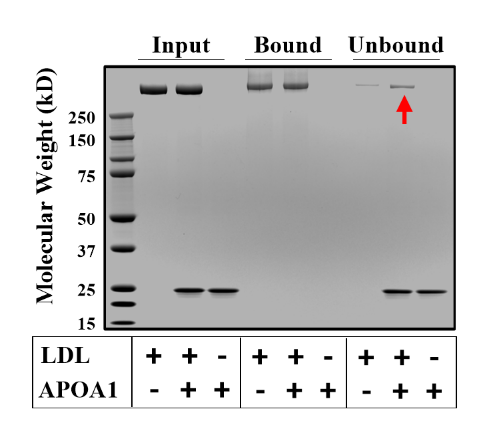
**SUPPLEMENTAL FIGURES**

***Supplemental Figure S1*. *APOA1 interferes with LDL binding to Chondroitin Sulfate.*** Chondroitin sulfate-coated beads were used to assess LDL binding in the presence and absence of APOA1. A CBB-stained gel of the pulled-down complexes reveals a higher amount of APOB (*red arrow*) in the unbound fractions when APOA1 was present, indicating reduced LDL binding.


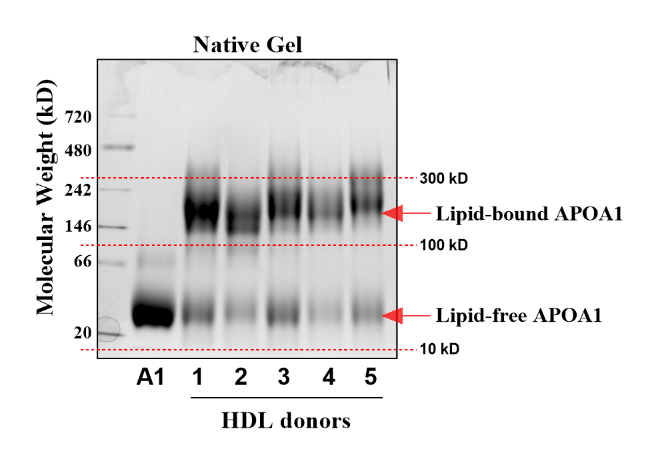
***Supplemental Figure S2*. Blue Native Gel Analysis of HDL:** Ultracentrifugally isolated HDL from five individuals was analyzed by BN-PAGE. Two prominent bands were observed: a larger band corresponding to the lipidated form of HDL, migrating just below the 242 kD marker, and a smaller band co-migrating with recombinant APOA1, appearing just above the 20 kD marker. *Abbreviation: A1, recombinant APOA1; kD, kilodalton.*


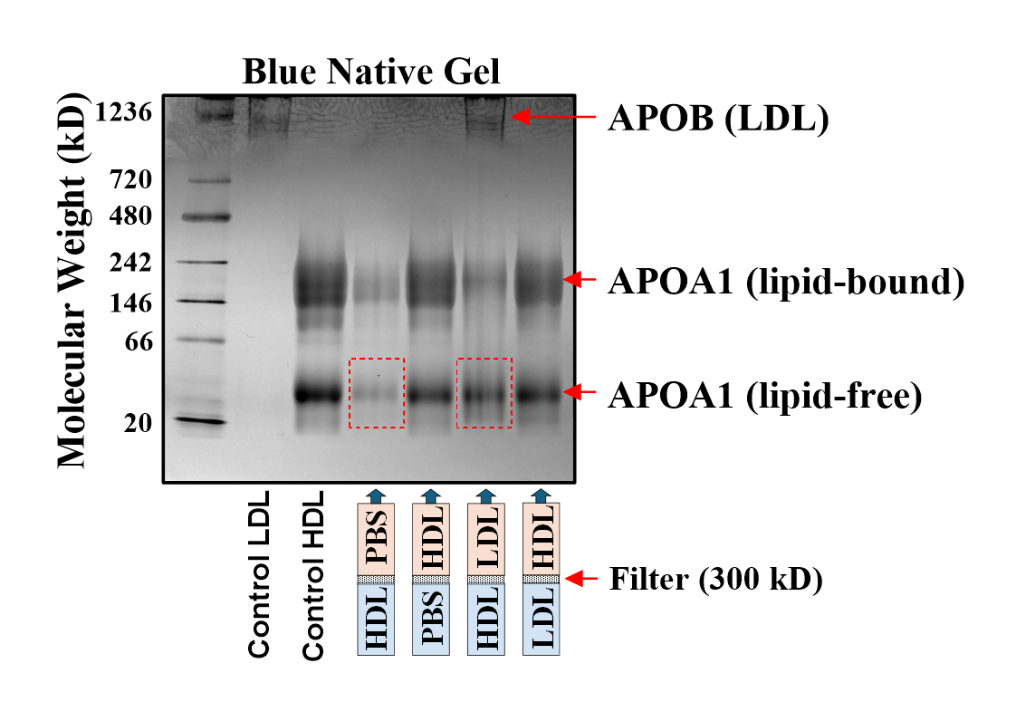
***Supplemental Figure S3.*** Native gel analysis of dialysates collected from LDL and PBS chambers (Chamber 1, *Top*) after equilibrium dialysis against HDL (Chamber 2, *Bottom*) shows a higher abundance of lipid-free APOA1 in the LDL dialysate compared to the PBS dialysate.
